# Liver Disease in Burn Injury: Evidence From a National Sample of 31,338 Adult Patients

**Published:** 2007-06-12

**Authors:** Leigh Ann Price, Brett Thombs, Catherine L. Chen, Stephen M. Milner

**Affiliations:** The Johns Hopkins Burn Center, Baltimore, Maryland

## Abstract

**Objective:** To assess mortality risk and extent of increased length of hospital stay in patients with burn injury with preexisting liver disease. **Methods:** Records of 31,338 adults who were admitted with burns to 70 burn centers were reviewed from the American Burn Association National Burn Repository. Demographics, percentage burn, and medical characteristics of 180 patients with liver disease were compared with all patients without liver disease and to a propensity score–matched sample of 180 patients without liver disease. Risk of mortality as well as lengths of both intensive care and total stay were compared after matching for demographics, burn injury, and preexisting medical conditions. **Results:** Patients with liver disease were significantly more likely to have a history of a number of medical comorbidities, including alcohol abuse, drug abuse, a psychiatric diagnosis, chronic pulmonary disease, hypertension, and diabetes. Patients with liver disease were significantly more likely to die in the hospital (27.2% vs 6.9%, odds ratio = 5.0, 95% confidence interval = 3.6–7.0, *P* < .01), and this held even when compared with a propensity score–matched group of patients without liver disease, but with similar demographics, burn injury, and medical profiles. Lengths of both intensive care and total hospital stay were 122.5% (*P* < .01) and 86.7% (*P* < .01) longer, respectively, among patients with liver disease than among all other patients. In a matched sample, lengths of both intensive care and total stays were longer, albeit not significantly so (41.6%, *P* = .12; 35.5%, *P* = .07). **Conclusions:** Liver impairment worsens the prognosis in patients with thermal injury.

Several preexisting medical conditions have been linked to increased mortality risk among burn patients. No studies, however, have assessed the impact of liver disease in the burn population. In addition, no statistical data exist to examine the effects of liver impairment on burn mortality or the relationship between this disease and length of care. A whole range of disorders of the liver, including portal hypertension, ascites, hepatic encephalopathy, jaundice, hepatorenal syndrome, and hepatitis, may influence the outcome following a burn injury. All have the potential to be life-threatening. We analyzed data from the American Burn Association National Burn Repository (ABA-NBR) to address specifically the effects of hepatic impairment on burn mortality and length of stay.

Our experience with patients with cirrhosis in the trauma and general surgical populations indicates increased risk of mortality with liver failure as predicted by the Child-Pugh classification. In a comprehensive analysis of 100 patients with cirrhosis undergoing abdominal operations, Garrison et al^1^ found that emergency operations were associated with more than a 5-fold increase (57% vs 10%) in mortality rate than in patients having elective operations. Doberneck et al^2^ reported similar observations, whereby a 4-fold increase in mortality rate following emergency procedures (45% vs 11%) was obtained. Garrison et al^1^ found that when patients were segregated into three groups according to the Child-Pugh classification, the mortality rates were 10% in class A, 31% in class B, and 76% in class C patients (Table 1). Age, burn size, and inhalation injury are robust predictors of mortality and of hospital stay following an acute burn injury.^3^–^6^

## METHODS

### Patient sample, variables, and outcome measures

Data were extracted from the ABA-NBR database for all adult patients aged 18 to 89 years who were admitted to 70 burn centers across the United States with thermal injuries (flame, contact, or scald) from 1995 through 2005. Patient data in the ABA-NBR included age, sex, race, year of injury, hospital admission status (eg, direct admission or transfer), the etiology and circumstances of the burn injury, mortality status and cause, percentage total body surface area (TBSA) burned, percentage TBSA burned second degree, percentage TBSA burned third degree, the presence or absence of an inhalation injury, and preexisting medical conditions.

In this study, patients with and without liver disease were compared and matched on the basis of demographics, burn injury, and preexisting medical conditions. Liver disease and other preexisting conditions were defined as clinical conditions that predated the burn injury and admission to the hospital and that could reasonably be expected to impact mortality and course of treatment.^7^ There was no limit to the number of different medical comorbidities that could be coded in the database for a given patient. Diagnosis codes for liver disease and other relevant preexisting medical conditions were selected from those included in the Deyo adaptation^8^ of the Charlson Index^9^ and the Elixhauser method of comorbidity measurement.^7^ The list of preexisting medical conditions included in this study and their ICD-9 codes are shown in Table 2.

Patients aged 90 and older were not included in the study because their actual ages were not entered into the dataset owing to patient privacy regulations. Patient records with missing data points were excluded from the analysis. In addition, all duplicate cases were removed from the dataset. A more complete description of the ABA-NBR database is provided elsewhere.^10^ Outcome measures were in-hospital death and, among survivors, lengths of both total hospital stay and intensive care stay.

### Statistical analysis

Statistical significance was defined as a probability value less than 0.05. χ^2^ and Fisher's exact tests were used to compare categorical variables, and 2-tailed *t* tests were used to compare continuous demographic and clinical variables between patients with liver disease and patients with no diagnosis of liver disease.

To adjust for differences in demographics, burn injury, and clinical characteristics of patients with liver disease and patients with no diagnosis of liver disease, we used a propensity score approach to generate a set of matched cases.^11^,^12^ The propensity score is a measure of the likelihood that a patient had a diagnosis of liver disease on the basis of relevant demographics, burn injury, and clinical variables. This approach reduces many covariates to a single variable, the propensity score. Since patients with similar propensity scores have similar characteristics, once matched, mortality and length of stay can be compared between patients with a diagnosis of liver disease and patients without a history of liver disease.

A stepwise logistic regression approach with *P* < .50 as the limit for variable entry was used to model the likelihood that patients had a diagnosis of liver disease on the basis of demographics, burn injury, and clinical variables. A propensity score was generated for each patient, and each patient who had a diagnosis of liver disease was matched with a patient with the closest possible propensity score who did not have a history of liver disease. The maximum allowable difference between propensity scores for matching was set at 0.1. When 2 or more patients without a history of liver disease had an identical propensity score, the matched patient was chosen randomly.^13^

Risk for death was assessed by calculating odds ratios for patients diagnosed with liver disease compared with propensity score–matched patients without a diagnosis of liver disease. Total length of hospital stay and the length of intensive care were positively skewed, and thus compared between patients with liver disease and matched controls using a nonparametric statistical test, the Wilcoxon rank sum test. All analyses were performed with SPSS, Version 14.0 (Chicago, Ill). Data are reported in the text as means (± standard deviation) or as odds ratios with 95% confidence intervals.

## RESULTS

### Patient characteristics

A total of 31,338 adult patients aged 18 to 89 were admitted to 70 burn centers. The mean age of adult patients in the ABA-NBR was 43.4 ± 17.2 years (range = 18–89 years); 72.8% of patients were men, and 65.7% were white. The mean percentage TBSA burned was 13.1% ± 16.2% (range = 0%–100%), and 13.2% of the sample incurred an inhalation injury. Table 2 shows the diagnoses of patients classified as having liver disease. The most common diagnoses were hepatitis of any variety (49.4%) and alcoholic cirrhosis (34.4%).

Baseline demographics, burn injury, and clinical characteristics of the patients by hepatic disease status are shown in Table 3. More than 50% of patients with liver disease also had a history of alcohol abuse and almost 20% had a history of drug abuse, compared with only 5.5% and 3.2%, respectively, in patients without a diagnosis of liver disease. Patients with hepatic insufficiency were also significantly more likely to have diagnoses of cardiac arrhythmias, cerebrovascular disease, chronic pulmonary disease, congestive heart failure, diabetes mellitus, HIV/AIDS, hypertension, and hypothyroidism. In addition, patients with liver disease were significantly older and had more extensive burn injuries.

The final propensity score model for matching patients included 12 individual predictor variables (age, TBSA burned third degree, congestive heart failure, hypertension, neurological disorder, chronic pulmonary disease, diabetes mellitus, peptic ulcer disease, obesity, alcohol abuse, a psychiatric diagnosis, and HIV/AIDS) and 10 interactions with age (congestive heart failure, neurological disease, chronic pulmonary disease, diabetes mellitus, peptic ulcer disease, obesity, alcohol abuse, drug abuse, a psychiatric diagnosis, and HIV/AIDS). The c-statistic for the propensity score model was 0.88, indicating adequate discrimination. Collinearity analysis showed that the variables included in the propensity score were not highly correlated; the highest bivariate correlation between any two variables was 0.35. All 180 patients with a diagnosis of liver disease were successfully matched: 85 on 5 or more digits of the propensity score, 65 on 4 digits, 21 on 3 digits, 8 on 2 digits, and 1 on 1 digit. After propensity score matching, there were no significant difference between the 180 patients with liver disease and the comparison group of 180 patients with no history of liver disease (Table 4).

### In-hospital mortality and length of stay

Prior to adjustment through matching, patients with a diagnosis of liver disease were almost 4 times as likely to die from the burn injury than patients without liver disease (27.2% vs 6.9%, *P* < .01). Even after matching across age, sex, burn injury severity, and other preexisting medical conditions, mortality rates were significantly higher among patients with liver disease than among matched controls (15.0%, *P* < .01). Mean length of intensive care was more than double for patients with liver disease (10.6 days) than for all patients without liver disease (4.5 days, *P* < .01), and total stay was almost 90% longer (18.8 days vs 12.9 days, *P* < .01). After matching, ICU stay was 41.6% longer for patients with live disease (*P* = .12) and total stay was 35.5% longer (*P* = .07) (Table 5).

## DISCUSSION

This is the first study to examine the association of the presence of liver disease with mortality risk and length of stay among patients with burn injury. Patients with a diagnosis of liver disease were significantly more likely than all other patients to die and required significantly longer lengths of both intensive care and total stay. Even when compared with a set of matched patients without liver disease, but who were similar in terms of demographics, burn injury, and medical profiles, patients with hepatic disease were significantly more likely to die from their burn injuries. Although not statistically significant, time in intensive care and total time in the hospital were also substantially longer among patients with liver disease than among matched controls.

Alteration of liver function tests is extremely common following major burns. An incidence as high as 50% has been described when there is transient elevation of the aspartate aminotransferase, alanine aminotransferase, and particularly the alkaline phosphatase. The precise etiology of these changes is not known, but they usually are benign in character and resolve spontaneously. Elevation of the serum bilirubin is somewhat less common but of more serious consequence. Patients who show severe and persistent derangements of the liver enzymes, including elevation of bilirubin, usually have some associated complication of their burns, such as burn wound sepsis, and in these patients, the prognosis is guarded.

### Effect of hepatic failure on burn pathophysiology

The liver plays an important role in the body's response to thermal injury. It is the principal organ responsible for producing acute-phase proteins and modulating the systemic inflammatory response.^14^ Following thermal injury, the acute-phase response brings about the activation of the coagulation and complement cascades, granulocytes, and monocytes/macrophages as well as platelets,^15^–^19^ and induces the liver to synthesize and release proteins that exert effects on a variety of tissues.^20^ After major trauma, such as a severe burn, hepatic protein synthesis shifts from hepatic constitutive proteins, such as albumin, prealbumin, transferrin, and retinol-binding protein, to acute phase proteins,^14^ which serve as mediators of the inflammatory process, function as transport proteins, and participate in burn wound healing.^21^,^22^ Jeschke et al^23^ recently demonstrated that the acute-phase response persists for longer duration than previously thought, which suggests that any compromise in liver function as seen in patients with chronic liver disease has both short-term and long-term detrimental effects on the body's ability to respond to burn injury.

### Impaired glucose tolerance

The liver helps maintain glucose homeostasis by synthesizing glycogen and generating glucose from precursors. Glucose metabolism in patients with severe burn is almost always abnormal. Patients that are victims of sepsis, burn, or trauma commonly enter a hypermetabolic stress state that is associated with changes in carbohydrate metabolism, such as enhanced peripheral glucose uptake and utilization, hyperlactatemia, increased glucose production, depressed glycogenesis, glucose intolerance, and insulin resistance.^24^ These abnormalities would likely be exaggerated in patients with liver disease, as altered glucose tolerance is known to occur in alcoholic chronic liver disease, nonalcoholic fatty liver, chronic hepatitis C, and portal hypertension.^25^ The impaired metabolism of glucose is aggravated with the progression of chronic hepatitis to liver cirrhosis.^25^ In patients with cirrhosis, the prevalence of impaired glucose tolerance is estimated to be about 60% to 80%, whereas that of overt diabetes is about 7% to 15%.^25^

### Reduced absorption of fat-soluble vitamins and nutrients

The liver is vital to the uptake, storage, and mobilization of vitamins, which play a significant role in wound healing, energy, metabolism, inflammation, and antioxidant activity.^14^ The fat-soluble vitamins, Vitamins A, E, D, and K, the absorption of which are dependent on bile salts, are the most important in relation to thermal injury.^14^ A failure to secrete bile leads to poor solubilization of dietary lipids and the fat-soluble vitamins, resulting in malabsorption and deficiency states, respectively.^26^ Malnutrition is common in patients with advanced cirrhosis and is associated with increased infection risk, multiorgan complications, hemorrhage from esophageal varices, posttransplant infection, prolonged hospitalization, and mortality.^27^ Postburn hypermetabolism frequently causes a vitamin deficiency, requiring supplementation to facilitate recovery.^28^ These data suggest that poor nutritional status arising from chronic liver disease prior to burn injury could complicate the repletion of essential nutrients in the postburn period and directly increase the risks of morbidity and mortality following burn injury.

### Drug metabolism and detoxification

Many liver diseases significantly compromise drug metabolism by impairing cytochrome P450 and other important hepatic enzymes. As a result of this slowed metabolism, the levels of the active forms of many drugs may be significantly higher than intended, and thereby cause toxic effects. Conventional wisdom dictates that the doses of many drugs be lowered for individuals with hepatic disease. However, burn injury induces many pathological changes in the human body, leading to alterations in pharmacokinetic parameters such as bioavailability, protein binding, volume of distribution (*V*_*d*_), and clearance.^29^ The pharmacokinetic parameters following burn injury vary between the acute phase of injury and the second hypermetabolic phase beyond the initial 48 hours of thermal injury.^30^ Decreased blood flow to tissues during the resuscitation phase reduces the rate of drug elimination by the kidneys and liver.^30^ During the hypermetabolic phase, increased blood flow may increase enough clearance of some of the drugs in order to require higher dosages than usual.^30^ The free fraction of a drug in plasma may be increased or decreased depending on its protein-binding characteristics.^29^,^31^ In addition, thermal injury has a direct impact on hepatic metabolism: the rate of phase I metabolism decreases, which is thought to be related to the oxygen-derived free radicals released during the course of injury, whereas phase II metabolism is unimpaired and may possibly increase.^30^ Other liver functions, such as protein synthesis, can also be disrupted by thermal injury.^30^ Given the extreme interpatient variability in drug handling among patients with burn injury, particularly with regards to antibiotics,^32^ preexisting liver disease can further complicate the dosing of drugs administered in the postburn period. Weinbren cautions that some patients may be exposed to subtherapeutic levels of antibiotics due to its rapid clearance even if the dose provided is perceived to be sufficiently in excess of requirement.^32^ Weinbren's experience with antibiotics in patients with burn injury suggests that, in general, one must balance the need to increase dosing to achieve therapeutic drug levels with the need to avoid toxicity in a patient with chronic liver disease.

In addition, the capacity of liver to detoxify exogenous and endogenous chemicals, foreign molecules, and hormones is limited by chronic liver disease. Clearance of bacteria by Kupffer cells of the liver normally helps prevent gut-derived bacteria from entering the systemic circulation. Loss of this function in liver disease as a result of portal-to-systemic shunting may help explain why patients with severe liver disease are more susceptible to developing systemic infections and septic complications.^33^ In their review article, Gosain and Gamelli^34^ state that there is a loss of physical barrier function in the gastrointestinal tract after burn injury, allowing translocation of bacteria and endotoxin to the portal circulation, which can lead to the development of multiple-organ failure. Most burn-related deaths (54%) in modern burn units occur because of septic shock and organ dysfunction.^35^ The loss of the liver's ability to clear gut-derived bacteria most likely increases the risk of postburn sepsis in these patients.

### Coagulation deficiency

Thrombotic and fibrinolytic mechanisms are activated after burn injury, with the extent of activation correlated to the severity of the thermal injury.^14^ Although much attention has been placed on increased thrombogenicity in patients with burn injury resulting from a decrease in antithrombin III, protein C, and protein S levels, complications of hypercoagulability in such patients are rare.^36^ Barret and Gomez^37^ have also suggested that the true incidence of disseminated intravascular coagulation, documented in up to 30% of examined cases by Wells et al, is much lower than previously reported. In contrast, patients with burn injury with preexisting liver disease have increased risk of hemorrhagic complications. The liver produces most of the factors involved in coagulation and fibrinolysis that helps maintain the balance between thrombin deposition and removal.^38^ Patients with cirrhosis frequently experience hemostatic disorders caused by abnormalities of platelet number and/or function, increased fibrinolysis, and deficient synthesis of clotting factors.^38^ Clotting factor deficiencies develop with the loss of functioning liver paranchymal cells, with early deficits in the Vitamin K–dependent factors (II, VII, IX, X, protein S, and protein C).^38^ The loss of factor VII significantly interferes with the function of the extrinsic coagulation cascade.^38^ Hemorrhagic complication is a major concern in patients with cirrhosis undergoing surgical interventions^39^ and must be considered when preparing a patient with burn injury with preexisting liver disease for surgery.

Traditionally, patients with liver disease have been considered to be at high risk for mortality, particularly in the context of patients with liver failure. It is not surprising that individuals with hepatic insufficiency are at an increased likelihood of dying from the burn injury in comparison with those without liver disease. These patients tend to have multiple medical problems coexisting with and stemming from their damaged liver. Two factors that often contribute to mortality are the propensity to bleed because of coagulopathies and ascites, which often lead to sepsis. The physiologic stress placed on patients with burn injury along with their hypermetabolic demands may lower the threshold for survival.

There are limitations that should be taken into consideration while interpreting the results from this study. The data are from a large registry and are to some degree flawed in their ability to capture patient illness severity. Patient comorbidity and injury variables, such as TBSA burned or the presence or absence of inhalation injury, were extracted by chart review and diagnoses were documented with ICD-9 coding rather than by more precise methods. An additional limitation is that differences in mortality across burn care centers related to differences in standard burn management or differences in patient characteristics were not explicitly incorporated into the analysis. The ABA-NBR does not include data on important factors that may differ across centers, such as time from burn to admission or fluid resuscitation. To the extent that a large number of burn centers were included in this study, however, it is not unreasonable to think that the results of this study are representative of typical patterns across burn centers in the United States.

In summary, we found that patients with a diagnosis of liver disease are at substantially greater risk for mortality subsequent to burn injury, even after matching on key demographics, burn injury, and medical variables. In addition, patients with liver disease who survive their injuries tend to require longer hospital stays than their matched counterparts without liver disease.

## Figures and Tables

**Table 1:** The Child-Pugh classification for assessing the severity of liver cirrhosis*

Score	1	2	3
Total bilirubin, mg/dL	<2.0	2.0–3.0	>3.0
Albumin, g/dL	>3.5	2.8–3.5	<2.8
Prothrombin time (prolonged), s, or prothrombin time INR	<4	4–6	>6
	<1.7	1.7–2.3	>2.3
Encephalopathy	None	Mild	Moderate
Ascites	None	Mild	Moderate

*The individual scores are summed and then grouped as follows: A = 5–6; B = 7–9; and C = 10–15. A “C” classification forecasts a survival of less than 12 months. INR indicates international normalized ratio.

**Table 2:** ICD-9-CM codes of comorbidities

**Comorbidity**	**ICD-9-CM codes**
Alcohol abuse	291.1, 291.2, 291.5, 291.8, 291.9, 303.90–303.93, 305.00–305.03, V113
Cardiac arrhythmias	426.10, 426.11, 426.13, 426.2–426.53, 426.6–426.89, 427.0, 427.2, 427.31, 427.60, 427.9, 785.0, V45.0, V53.3
Chronic pulmonary disease	490–496, 500–505, 506.4
Congestive heart failure	398.91, 402.11, 402.91, 404.11, 404.13, 404.91, 404.93, 428.0–428.9
Dementia	290–290.9
Diabetes	250–250.33, 250.40–250.73, 250.90–250.93
Drug abuse	292.0, 292.82–292.89, 292.9, 304.00–304.93, 305.20–305.93
HIV/AIDS*	042
Hypertension	401.1, 401.9, 402.10, 402.90, 404.10, 404.90, 405.11, 405.19, 405.91, 405.99
Hypothyroidism	243–244.2, 244.8, 244.9
Cerebrovascular disease	438.0
Liver disease	070.32, 070.33, 070.54, 456.0, 456.1, 456.20, 456.21, 571.0, 571.2, 571.3, 571.40–571.49, 571.5, 571.6, 571.8, 571.9, 572.2–572.8, V42.7
Metastatic cancer†	196.0–199.1
Nonmetastatic malignancy†	140–172.9, 174–195.8, 200–208.9, 238.6, 273.3, V10.00–V10.9
Obesity	278.0
Old myocardial infarction	412.0
Other neurological disorders	331.9, 332.0, 333.4, 333.5, 334.0–335.9, 340, 341.1–341.9, 345.00–345.11, 345.40–345.51, 345.80–345.91
Paralysis	342.0–342.12, 342.9–344.9
Peptic ulcer disease	531–534.9, V12.71
Peripheral vascular disease	440.0–440.9, 441.2, 441.4, 441.7, 441.9, 443.1–443.9, 447.1, 557.1, 557.9, V43.4
Psychiatric diagnosis	295.0–295.9, 296.0, 296.2–298.9
Pulmonary circulation disorders	416.0–416.9, 417.9
Renal disease	403.11, 403.91, 404.12, 404.92, 582–582.9, 583–583.7, 585, 586, 588–588.9, V42.0, V45.1, V56.0, V56.8
Rheumatologic disease	701.0, 710.0–710.9, 714.0–714.9, 720.0–720.9, 725
Valvular disease	093.20–093.24, 394.0–397.1, 424.0–424.91, 746.3–746.6, V42.2, V43.3

*Excludes asymptomatic HIV infection status.

^†^If both solid tumor without metastatis and metastatic cancer are present, only metastatic cancer is counted.

**Table 3:** Diagnoses of patients with liver disease

**Diagnosis**	Number (%)* of patients with liver disease
070.32 Hepatitis B	11 (6.1)
070.54 Hepatitis C	66 (36.7)
456.0, 456.1 Esophageal varices	16 (8.9)
571.2, 571.3 Alcoholic cirrhosis	62 (34.4)
571.3, 571.4–571.49 Chronic hepatitis	16 (8.9)
571.5, 571.8, 571.9 Chronic liver disease, no mention of alcohol	25 (13.9)
572.2 Hepatic coma	6 (3.3)
572.3 Portal hypertension	7 (3.9)
572.4 Hepatorenal syndrome	5 (2.8)
572.8 Other sequelae of chronic liver disease	16 (8.9)

*Percentages do not add to 100% owing to multiple diagnoses related to liver disease in some patients.

**Table 4:** Baseline demographic and clinical characteristics*

		**Prepropensity score matching**		**Postpropensity score matching**	
			
	**Liver disease (*n* = 180)**	**No liver disease (*N* = 31,158)**	***P***	**No liver disease (*n* = 180)**	***P***
Age, mean ± SD, y	51.4 ± 11.8	43.3 ± 17.3	<.01	50.2 ± 14.2	.38
Total TBSA, mean ± SD, %	17.1 ± 16.9	13.1 ± 16.1	<.01	17.1 ± 18.7	.99
FT-TBSA, mean ± SD, %	9.6 ± 15.8	5.1 ± 13.1	<.01	10.0 ± 17.12	.83
Inhalation injury	45 (25.0)	4,101 (13.2)	<.01	40 (22.2)	.54
Fire/flame or inhalation	136 (75.6)	2,0125 (64.6)	<.01	138 (76.7)	.81
Male gender	127 (70.6)	22,698 (72.8)	.49	116 (64.4)	.22
White	116 (66.3)	19,844 (65.7)	.87	115 (66.9)	.91
Alcohol abuse	94 (52.2)	1,720 (5.5)	<.01	101 (56.1)	.46
Cardiac arrhythmias	10 (5.6)	610 (2.0)	<.01	7 (3.9)	.46
Cerebrovascular disease	3 (1.7)	104 (0.3)	.02	1 (0.6)	.31
Chronic pulmonary disease	43 (23.9)	1,560 (5.0)	<.01	43 (23.9)	1.00
Congestive heart failure	11 (6.1)	476 (1.5)	<.01	8 (4.4)	.48
Dementia	0 (0.0)	87 (0.3)	.61	0 (0.0)	1.00
Diabetes mellitus	23 (12.8)	1,342 (4.3)	<.01	21 (11.7)	.75
Drug abuse	34 (18.9)	987 (3.2)	<.01	29 (16.1)	.49
HIV/AIDS	7 (3.9)	61 (0.2)	<.01	5 (2.8)	.56
Hypertension	45 (25.0)	2,949 (9.5)	<.01	42 (23.3)	.71
Hypothyroidism	6 (3.3)	268 (0.9)	<.01	6 (3.3)	1.00
Metastatic cancer	0 (0.0)	46 (0.1)	.77	1 (0.6)	.50
Nonmetastatic malignancy	0 (0.0)	137 (0.4)	.45	2 (1.1)	.25
Obesity	5 (2.8)	376 (1.2)	.07	3 (1.7)	.36
Old myocardial infarction	5 (2.8)	259 (0.8)	.02	7 (3.9)	.56
Other neurological disorders	8 (4.4)	479 (1.5)	<.01	8 (4.4)	1.00
Paralysis	1 (0.6)	214 (0.7)	.65	0 (0.0)	.50
Peptic ulcer disease	9 (5.0)	120 (0.4)	<.01	6 (3.3)	.43
Peripheral vascular disease	3 (1.7)	190 (0.6)	.10	2 (1.1)	.50
Psychiatric diagnosis	22 (12.2)	874 (2.8)	<.01	27 (15.0)	.44
Pulmonary circulation disorders	0 (0.0)	54 (0.2)	.73	1 (0.6)	.50
Renal disease	4 (2.2)	173 (0.6))	.02	4 (2.2)	1.00
Rheumatologic disease	3 (1.7)	125 (0.4)	.03	1 (0.6)	.31
Valvular disease	0 (0.0)	107 (0.3)	.51	2 (1.1)	.50

*Values given are number (percentage) unless otherwise indicated. TBSA indicates total body surface area.

**Table 5:** Mortality and length of stay of patients with liver disease compared with all patients with no liver disease and with a matched sample of patients with no liver disease

	**No liver disease**			**Prematch**		**Postmatch**	
				
	**Prematch (*N* = 31,158)**	**Postmatch (*n* = 180)**	**Liver disease (*n* = 180)**	**Odds ratio (95% CI)**	***P***	**Odds ratio (95% CI)**	***P***
Mortality, *n* (%)	2154 (6.9)	27 (15.0)	49 (27.2)	5.0 (3.6–7.0)	≪.01	2.1 (1.3–3.6)	<.01
		**No liver disease after matching (*n* = 180)**	**Liver disease (*n* = 180)**	**% Increase prematch**	***P***	**% Increase postmatch**	***P***
Length of stay							
ICU, days (SD)	4.5 (13.9)	7.5 (16.9)	10.6 (21.4)	122.5	≪.01	41.6	.12
Total, days (SD)	12.9 (19.3)	18.8 (21.5)	25.4 (28.6)	86.7	<.01	35.5	.07

## References

[1] Garrison RN, Crver HM, Howard DA, Polk HC (1984). Clarification of risk factors for abdominal operations in patients with hepatic cirrhosis. Ann Surg.

[2] Doberneck RC, Sterling WA, Allison DC (1983). Morbidity and mortality after operation in nonbleeding cirrhotic patients. Am J Surg.

[3] Ryan CM, Schoenfeld DA, Thorpe WP, Sheridan RL, Cassem EH, Tompkins RG (1998). Objective estimates of the probability of death from burn injuries. N Engl J Med.

[4] Smith DL, Cairns BA, Ramadan F (1994). Effect of inhalation injury, burn size, and age on mortality: a study of 1447 consecutive burn patients. J Trauma.

[5] Griffe O, Gartner R, Captier G (2001). Evaluation of prognostic factors in the burned patient [in French]. Ann Chir Plast Esthet.

[6] O'Keefe GE, Hunt JL, Purdue GF (2001). An evaluation of risk factors for mortality after burn trauma and the identification of gender-dependent differences in outcomes. J Am Coll Surg.

[7] Elixhauser A, Steiner C, Harris DR, Coffey RM (1998). Comorbidity measures for use with administrative data. Med Care.

[8] Deyo RA, Cherkin DC, Ciol MA (1992). Adapting a clinical comorbidity index for use with ICD-9-CM administrative databases. J Clin Epidemiol.

[9] Charlson ME, Pompei P, Ales KL, MacKenzie CR (1987). A new method of classifying prognostic comorbidity in longitudinal studies: development and validation. J Chronic Dis.

[10] American Burn Association (2006). National Burn Repository: 2005 Report.

[11] D'Agostino RB (1998). Propensity score methods for bias reduction in the comparison of a treatment to a non-randomized control group. Stat Med.

[12] Rosenbaum PR, Rubin DB (1984). Reducing bias in observational studies using subclassification on the propensity score. J Am Stat Assoc.

[13] Parsons LS (2001). Reducing bias in a propensity score matched-pair sample using greedy matching techniques.

[14] Jeschke MG, Herndon DN (2002). The hepatic response to a thermal injury. Total Burn Care.

[15] Bartlett RH, Fong SW, Marrujo G, Hardeman T, Anderson W (1981). Coagulation and platelet changes after thermal injury in man. Burns.

[16] Eurenius K, Mortensen PF, Meserol PM, Curreri PW (1971). Platelet and megakaryocyte kinetics following thermal injury. J Lab Clin Med.

[17] Eurenius K, Rossi TD, McEuen DD, Arnold J, McManus WF (1974). Blood coagulation in burn injury. Proc Soc Exp Biol Med.

[18] Emerson WA, Leve PD, Krevans JR (1970). Hematologic changes in septicemia. Johns Hopkins Med J.

[19] Xia ZF, Coolbaugh MI, He F, Herdon DN, Papaconstantinou J (1992). The effects of burn injury on the acute phase response. J Trauma.

[20] Kushner I (1982). The phenomenon of the acute phase response. Ann N Y Acad Sci.

[21] Tilg H, Dinarello CA, Mier JW (1997). IL-6 and APPs: anti-inflammatory and immunosuppressive mediators. Immunol Today.

[22] Jeschke MG, Herndon DN, Wolf SE (1999). Recombinant human growth hormone alters acute phase reactant proteins, cytokine expression, and liver morphology in burned rats. J Surg Res.

[23] Jeschke MG, Barrow RE, Herndon DN (2004). Extended hypermetabolic response of the liver in severely burned pediatric patients. Arch Surg.

[24] Mizock BA (1995). Alterations in carbohydrate metabolism during stress: a review of the literature. Am J Med.

[25] Picardi A, D'Avola D, Gentilucci UV (2006). Diabetes in chronic liver disease: from old concepts to new evidence. Diab Metab Res Rev.

[26] Henkel AS, Buchman AL (2006). Nutritional support in patients with chronic liver disease. Nat Clin Pract Gastroenterol Hepatol.

[27] Buchman AL (2006). Total parenteral nutrition: challenges and practice in the cirrhotic patient. Transplant Proc.

[28] Klein GL, Herndon DN (2002). Vitamin and trace element homeostasis following severe burn injury. Total Burn Care.

[29] Jaehde U, Sorgel F (1995). Clinical pharmacokinetics in patients with burns. Clin Pharmacokinet.

[30] Bonate PL (1990). Pathophysiology and pharmacokinetics following burn injury. Clin Pharmacokinet.

[31] Martyn JA, Abernethy DR, Greenblatt DJ (1984). Plasma protein binding of drugs after severe burn injury. Clin Pharmacol Ther.

[32] Weinbren MJ (1999). Pharmacokinetics of antibiotics in burn patients. J Antimicrob Chemother.

[33] Nguyen TT, Lingappa VR, McPhee SJ, Ganong WF (2006). Liver disease. Pathophysiology of Disease: An Introduction to Clinical Medicine.

[34] Gosain A, Gamelli RL (2005). Role of the gastrointestinal tract in burn sepsis. J Burn Care Rehabil.

[35] Church D, Elsayed S, Reid O, Winston B, Lindsay R (2006). Burn wound infections. Clin Microbiol Rev.

[36] Barret JP, Dziewulski PG (2006). Complications of the hypercoagulable status in burn injury. Burns.

[37] Barret JP, Gomez PA (2005). Disseminated intravascular coagulation: a rare entity in burn injury. Burns.

[38] Fitz JG, Feldman M, Friedman LS, Brandt LJ (2006). Hepatic encephalopathy, hepatopulmonary syndromes, hepatorenal syndrome, and other complications of liver disease. Sleisenger and Fordtran's Gastrointestinal and Liver Disease: Pathophysiology, Diagnosis, Management.

[39] Hayashida N, Aoyagi S (2004). Cardiac operations in cirrhotic patients. Ann Thorac Cardiovasc Surg.

